# Fluorescent Protein-Based Ca^2+^ Sensor Reveals Global, Divalent Cation-Dependent Conformational Changes in Cardiac Troponin C

**DOI:** 10.1371/journal.pone.0164222

**Published:** 2016-10-13

**Authors:** Myriam A. Badr, Jose R. Pinto, Michael W. Davidson, P. Bryant Chase

**Affiliations:** 1 Department of Biological Science, Florida State University, Tallahassee, Florida, United States of America; 2 Institute of Molecular Biophysics, Florida State University, Tallahassee, Florida, United States of America; 3 Department of Biomedical Sciences, College of Medicine, Florida State University, Tallahassee, Florida, United States of America; 4 National High Magnetic Field Laboratory, Florida State University, Tallahassee, Florida, United States of America; University of Cincinnati, UNITED STATES

## Abstract

Cardiac troponin C (cTnC) is a key effector in cardiac muscle excitation-contraction coupling as the Ca^2+^ sensing subunit responsible for controlling contraction. In this study, we generated several FRET sensors for divalent cations based on cTnC flanked by a donor fluorescent protein (CFP) and an acceptor fluorescent protein (YFP). The sensors report Ca^2+^ and Mg^2+^ binding, and relay global structural information about the structural relationship between cTnC’s N- and C-domains. The sensors were first characterized using end point titrations to decipher the response to Ca^2+^ binding in the presence or absence of Mg^2+^. The sensor that exhibited the largest responses in end point titrations, CTV-TnC, (Cerulean, TnC, and Venus) was characterized more extensively. Most of the divalent cation-dependent FRET signal originates from the high affinity C-terminal EF hands. CTV-TnC reconstitutes into skinned fiber preparations indicating proper assembly of troponin complex, with only ~0.2 pCa unit rightward shift of Ca^2+^-sensitive force development compared to WT-cTnC. Affinity of CTV-TnC for divalent cations is in agreement with known values for WT-cTnC. Analytical ultracentrifugation indicates that CTV-TnC undergoes compaction as divalent cations bind. C-terminal sites induce ion-specific (Ca^2+^ versus Mg^2+^) conformational changes in cTnC. Our data also provide support for the presence of additional, non-EF-hand sites on cTnC for Mg^2+^ binding. In conclusion, we successfully generated a novel FRET-Ca^2+^ sensor based on full length cTnC with a variety of cellular applications. Our sensor reveals global structural information about cTnC upon divalent cation binding.

## Introduction

Troponin C (TnC) is central to muscle regulation by calcium ion. TnC is part of the troponin ternary complex that consists of three distinct polypeptides [1]: troponin I (TnI), the inhibitory subunit; troponin T (TnT), which holds the complex on tropomyosin; and TnC, the Ca^2+^ sensing subunit in both cardiac and skeletal muscle. Its tertiary structure consists of two globular domains which are connected by a central helical linker. The C-domains of the cardiac and skeletal isoforms of TnC contain two EF-hand, divalent cation-binding sites (sites III and IV) that can bind either Ca^2+^ with a high affinity (2 x 10^7^ M^1^) or Mg^2+^ at lower affinity (3.5 x 10^3^ M^-1^) [2]. Sites III and IV are considered “structural sites” because the affinity of the C-domain for TnI is enhanced when these sites are occupied by divalent cations; physiologically, Mg^2+^ is thought to be bound at the C-terminal sites under diastolic conditions, but may be partially displaced by Ca^2+^ during systolic activation [3,4]. In the N-domain, the cardiac isoform of TnC (cTnC) binds Ca^2+^ at site II with a lower Ca^2+^ affinity (5 x 10^5^ M^-1^) [2]. N-terminal site II of cTnC is the regulatory trigger site because cardiac contraction is activated by Ca^2+^ binding at site II during systole. The primary sequence of site I within cTnC renders it evolutionarily non-functional for divalent cation binding [5]. Local structural changes have been extensively studied within the N- or C-domain upon divalent cation binding [6–11], but less is known about modulation of the global structure of TnC. Structure of TnC’s central helix linker differs between crystal [12–14] and solution [15–18] assays for TnC on its own. The structural relationship between the N- and C-domains appears to be dependent on whether Ca^2+^ is present and which isoform is being considered, and also the presence of other Tn subunits [6,7,18,19]. Distortion of the global structure of purified cTnC by formation of a disulfide bond between Cys 84 in the linker helix and Cys 35 in the N-domain is associated with constitutive, Ca^2+^-independent activation of muscle fibers when this modified protein is reconstituted into troponin complex on thin filaments [20]. Furthermore, several mutations have been identified in the TNNC1 gene that are associated with cardiomyopathies [21–28]; while many functional studies have been carried out using these mutants, it is not known whether they might affect the structural relationship between the N- and C-domains.

Structural changes in TnC are the basis for a recently developed family of genetically encoded Ca^2+^ indicators (GECI’s) [29,30]. Heim and Griesbeck [29] generated a GECI (TN-L15) based on chicken skeletal TnC (missing the N-terminal 14 amino acids) flanked by the FRET pair CFP and citrine (YFP). They also described a GECI in which chicken skeletal TnC was swapped with human cTnC (hcard-TnC). Mank et al. [30] further modified the biosensor to contain two copies of the C-terminus of chicken skeletal TnC (with no N-terminal domain), sandwiched between CFP and citrine (TN-XXL). For many applications, these constructs improved upon earlier calmodulin-based GECIs such as the FRET-sensor Cameleon [31], and single fluorophore GECIs, i.e., Camgaroos and Pericams [32,33].

We have designed GECI’s that are based on full length human cardiac TnC flanked by various improved FRET pairs of fluorophores. FRET utilizes the physical process by which non-radiative energy is transferred from a shorter wavelength donor chromophore to a longer wavelength acceptor chromophore through intermolecular, long-range dipole-dipole coupling [34]. Based on structural studies of cTnC [19,35] and the observation that cTnC is constitutively activated when a disulfide bond forms between Cys 35 in the N-domain and Cys 84 in the linker helix [20], we hypothesized that Ca^2+^-dependent compaction of cTnC would result in increased FRET between the fluorophores as the ends of cTnC come together. We also hypothesized that sites III and IV at the C-terminus of TnC induce global conformational changes upon binding Ca^2+^ or Mg^2+^. Conversely, in the absence of divalent cations, we hypothesized that the protein would adopt a more relaxed conformation, unfavorable for FRET. The first aspect of our study is aimed at understanding more about the functional and structural changes of human cardiac TnC, upon binding of divalent cations. Understanding these conformational changes will pave the way for understanding the effect of mutations on TnC and muscle contraction. The second aspect of the project aims at using the Ca^2+^ dependent FRET TnC as a sensor for Ca^2+^. This construct has the potential to be used as a Ca^2+^ sensor in the non-muscle field, but perhaps the most exciting use highlighted by this study is reporting on Ca^2+^ in the muscle: TnC-extracted skinned fibers could be reconstituted with the construct and retained all the contractile properties of the cardiac muscle.

## Materials and Methods

### Generation of Fluorescent Constructs

**Fig 1:**
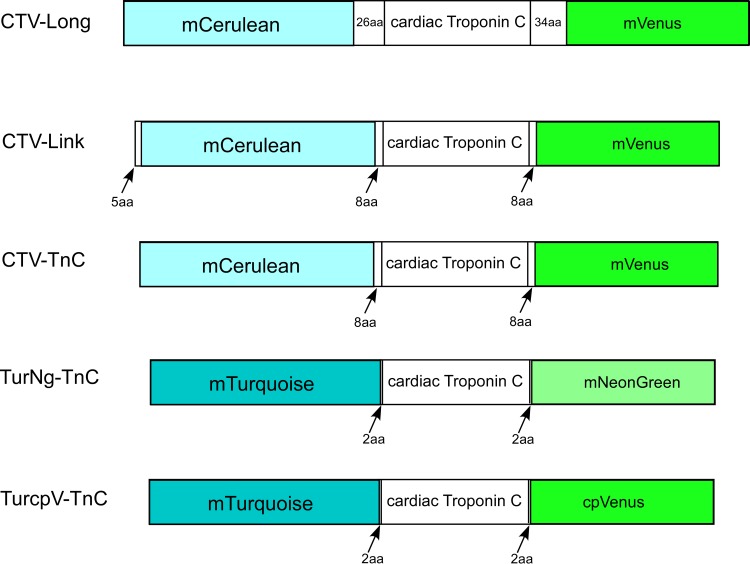
FRET-TnC constructs varying the FRET pair of fluorophores and linker length. Five constructs were generated by flanking human cardiac troponin C (cTnC) with one of three FRET pairs of fluorophores. Constructs also differ by the linker length (indicated by number of amino acids, aa) separating the fluorescent proteins from cTnC.

Plasmids encoding the fluorescent proteins mCerulean (CFP, λ_ex_: 433nm, λ_em_: 475nm) [36], mVenus (YFP, λ_ex_: 515nm, λ_em_: 525nm) [37], mTurquoise (CFP, λ_ex_: 434nm, λ_em_: 474nm) [38], cpVenus (YFP, λ_ex_: 515nm, λ_em_: 525nm), and mNeonGreen (Y/GFP, λ_ex_: 506nm, λ_em_: 517nm) [39] were generated at Florida State University’s National High Magnetic Field Laboratory. Human cTnC (AAB91994) from a human cardiac cDNA library from BD bioscience (San Jose, California, USA) [40] was cloned into TOPO vector from Life Technologies (Grand Island, NY, USA). A total of five FRET constructs were generated (Fig 1). CTV-Long contains mCerulean and mVenus, flanking cTnC separated by 26 and 34 amino acid linkers (N-terminal linker “SGLRSRGNNSPLEIILFNFKKEISRR”, and C-terminal linker “RTGCNKARKEAELAAEGRIPAHWRPLLVDPPVAT”) respectively. Similarly, CTV-Link contains mCerulean and mVenus separated from cTnC with 8 amino acids (N-terminal linker “SGLRSRGG” and C-terminal linker “GGDPPVAT”), also containing an extra 5 amino acids at the N-terminus of mCerulean. CTV-TnC has the same sequence as CTV-Link excluding the 5 amino acids at the N-terminus. TurNg-TnC is made from mTurquoise and mNeonGreen, separated from cTnC by 2 amino acids (N-terminal linker “SG”, and C-terminal linker “TG”). Similarly, TurcpV-TnC also possesses the same 2 amino acid linkers (N-terminal linker “SG” and C-terminal linker “TG”), but the fluorescent proteins are mTurquoise and cpVenus. Finally, the control protein TV-Link (not shown) contains cTnC and mVenus separated by 8 amino acids.

**Fig 2:**
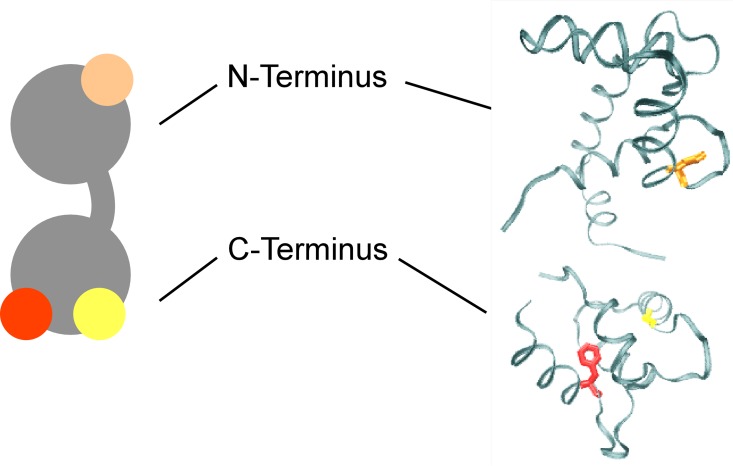
Schematic and crystal structure of cTnC. cTnC is shown in a schematic representation (gray, left) and the crystal structure (PDB 1J1E, right). Mutated residues (used in a subset of studies) are: D65 highlighted in orange at the N-terminus; and D104 and D140 highlighted in red and yellow, respectively, at the C-terminus.

We also generated multiple mutants of the construct CTV-TnC where one or more EF hand of TnC was rendered inactive, by substituting the first coordinating residue of the EF hand (Asp) with alanine, following the strategy of Putkey et al. [41] and as described by others [42–45]. The mutant with site III inactivated was D104A CTV, and the mutant with site IV inactivated was D140A CTV. A double mutant having both sites at the C-terminus mutated was D104-140A CTV. Finally, triple mutant 3XEF-CTV has all 3 sites inactivated (D65A-D104A-D140A) (Fig 2).

All constructs were expressed as GST fusion proteins in *E*. *coli* using vector pet41c, and purified on a GST-affinity column (GST-bind resin, EMD Millipore, Darmstadt, Germany), followed by an anion exchange column DE-52 (diethylaminoethyl cellulose, Whatman/GE healthcare, Wauwatosa, Wisconsin) after removal of GST with thrombin.

### Tn Subunits and Reconstitution of Tn Complex

Human cardiac troponin I (hcTnI (P19429, with all Cys residues mutated to Ala, named Cys-less TnI)) and human cardiac troponin T (hcTnT (P453796)) were kindly donated by Dr. Piotr Fajer (Institute of Molecular Biophysics, Florida State University). Proteins were expressed in BL21 *E*. *coli*. Recombinant TnI was purified using a weak cation exchange CM sepharose column (HiTrap CM FF, GE Healthcare, Fairfield, Connecticut, USA). Purification or recombinant TnT with an N-terminal His-tag began with a Ni column (Ni charged nitrilotriacetic acid, Amersham). The second purification step used a DE-52 anion exchange column after cleavage of the His-tag. The ternary troponin complex was reconstituted by combining the desired TnC construct with TnT and TnI at a molar ratio of 1:1:1.5. The proteins were mixed and dialyzed overnight against Reconstitution Buffer A (50mM MOPS, 0.5M KCl 1mM EDTA, 2mM CaCl_2_, 3mM MgCl_2_, 6M urea, pH = 7.2), followed by Reconstitution Buffer B (50mM MOPS, 0.5 M KCl, 2mM CaCl_2_, 3mM MgCl_2_, pH = 7.2). Finally, a two-step dialysis of 6hrs each against Reconstitution Buffer C (50mM MOPS, 0.1 M KCl, pH = 7.2) was performed. Reconstitution of the complex was evaluated by size exclusion chromatography (SEC; Superdex 200, 10/300 Cat. No. 17-5175-01, GE Healthcare Life Sciences).

### Ca^2+^ dependent FRET by End Point Titrations (+/- Mg^2+^)

End point titrations were used to evaluate changes in the FRET signal of the TnC fusion proteins upon binding of Ca^2+^ (in the presence or absence of Mg^2+^). These measurements involve 3-step titrations: in the initial condition, contaminating multivalent metal ions (particularly Ca^2+^) were chelated in the absence or presence of millimolar (physiological) levels of Mg^2+^ (100μM EDTA, or 100μM EGTA/3mM Mg^2+^); in step 2 we then switched to a high Ca^2+^ state (400μM Ca^2+^) which saturates all functional EF hands; and the third condition tests for reversibility of any changes by chelating Ca^2+^ added in step 2, in the absence or presence of Mg^2+^ (3mM EDTA or 3mM EGTA/3mM Mg^2+^ respectively). These experiments are considered end-point titrations because we are only titrating one ionic species in a single, large increase in concentration; this contrasts with full range titrations of selected TnCs described below. For end point titrations of Ca^2+^ in the absence of Mg^2+^, the protein was dialyzed against Titration Buffer EDTA (50mM MOPS, 0.1M KCl, 100μM EDTA, pH = 7.0). For end point titrations of Ca^2+^ in the presence of Mg^2+^, each of the constructs was dialyzed against Titration Buffer EGTA (50mM MOPS, 0.1M KCl, 100μM EGTA, 3mM MgCl_2_, pH 7.0). Proteins were diluted to 1μM. All fluorescence measurements for these experiments were performed using a spectrofluorometer (Varian Cary Eclipse, Agilent Technologies, Santa Clara Ca, USA). 30μl of sample at 1μM was placed in a small fluorescence cuvette (Starna Cell, 16.10 Q10). Data were collected with PMT excitation voltage of 700-800V, and T = 22°C. Ca^2+^ saturation was achieved by adding 2μL of 6mM CaCl_2_ to the cuvette to obtain a final concentration of 400μM Ca^2+^, which was chosen according to known affinities of TnC for Ca^2+^ [2].

**Fig 3:**
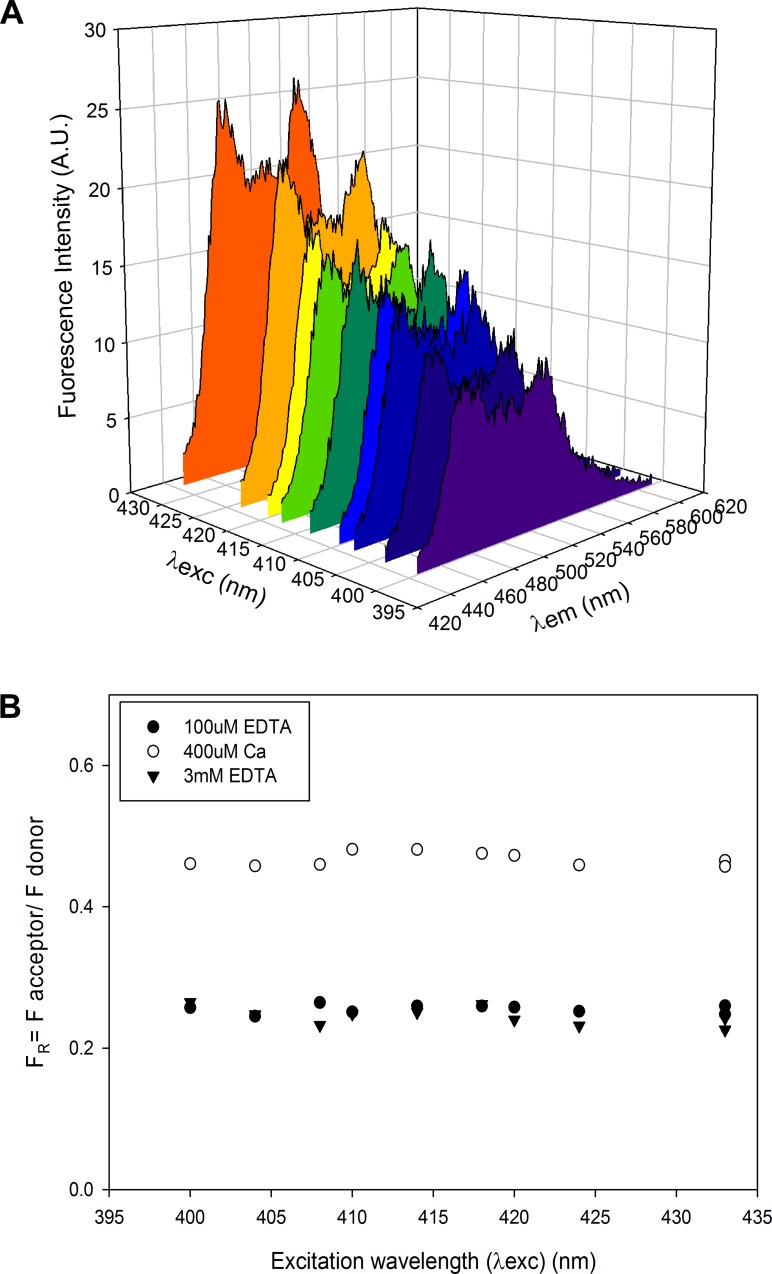
Examples of individual fluorescence spectra obtained during end point titrations. Examples of spectra (A) for construct CTV-TnC (1μM) at 400μM Ca total, excited at various excitation wavelengths (λ_ex_; from purple to orange, in order: 400nm, 404nm, 408nm, 410nm, 414nm, 418nm, 420nm, 424nm, 433nm). (B) FRET ratios (F_R_, Eq 2) plotted as a function of λ_ex_ for construct CTV-TnC (1μM), under the 3 conditions tested: initial condition 100μM EDTA (filled circles); next 400μM Ca (as in panel A) (open circles); and lastly 3mM EDTA (filled, inverted triangles). Note that F_R_ is independent of λ_ex_ and thus absolute value of acceptor emission intensity, and also that F_R_ varies reversibly with divalent cation binding.

For each of the three conditions, fluorescence emission spectra were obtained at several excitation wavelengths. For constructs containing mCerulean and mVenus (CTV-Long, CTV-Link, CTV-TnC), the initial measurement was made using an excitation wavelength λ_ex_ = 433nm, and collecting a fluorescence emission scan from 445-610nm. For proteins containing mTurquoise as the donor (TurNg-TnC, TurcpV-TnC), λ_ex_ = 434 nm, and the fluorescence emission scan was from 445-610nm. For each condition, we also collected several additional scans using a range of excitation wavelengths (λ_ex_ = 433nm, 424nm, 420nm, 418nm, 414nm, 410nm, 408nm, 404nm or 400nm) (Fig 3A) to ensure the validity of our analysis and its applicability to single molecule analyses: (i) to minimize bleedthrough of excitation, which is minimal; (ii) to reduce the non-FRET component of fluorescence emission resulting from direct excitation of the acceptor (at shorter λ_ex_, this effect is attenuated and the correction applied to obtain $F_{Acc}^{FRET}$ (Eq 1, described below) is smaller); and (iii) we are particularly interested in shorter λ_ex_ ~408nm because this wavelength is similar to laser excitation available for TIRF microscopy, allowing for a direct translation of the solution experiments to microscopy assays.

### End Point Titrations of Mg^2+^ for Mutants of CTV-TnC

Each construct was dialyzed against Titration Buffer EDTA (50mM MOPS, 0.1M KCl, 100μM EDTA, pH = 7.0) to chelate contaminating multivalent metal ions. Protein of interest was diluted to 1μM in Titration Buffer EDTA. 30μl sample at 1μM was placed in a small fluorescence cuvette (Starna Cell, 16.10 Q10) and data were collected as described above with PMT excitation voltage set to 700-800V, and T = 22°C. Emission scans were recorded from 445-610nm, with varying λ_ex_. This end-point titration is similar to that done with Ca^2+^, except that 10mM Mg (1μl of 300mM MgCl_2_) was added to saturate the protein, noting the known difference in affinities between Mg^2+^ and Ca^2+^. Divalent cations were subsequently chelated by adding 12mM EDTA (1μl of 360mM EDTA). Data were analyzed as described below.

### Analysis of Fluorescence Spectra for End Point Titrations

For each scan, the following analysis was applied to the spectrum to obtain an estimate of FRET occurring in each condition tested: *i)* Linear decomposition of each spectrum (scan from 445-610nm) yielding the individual contributions of the donor and acceptor. This allows separation of the donor and acceptor contributions at the peak wavelength for acceptor emission; *ii)* Correction for direct excitation of the acceptor (which was less at lower end of the range of λ_ex_ used in this study) to obtain acceptor fluorescence emission due to FRET only. Linear decomposition was achieved by minimizing the sum of squared residuals between an experimental spectrum and scaled versions of the appropriate donor and acceptor emission reference using a custom MatLab (The MathWorks, Inc., Natick, MA, USA) program developed by Dr. C. K. P. Loong. Linear decomposition yielded the peak amplitudes for donor fluorescence (*F*_*Don*_) and acceptor fluorescence (*F*_*Acc*_*)*. *F*_*Acc*_ contains two components, one due to FRET excitation ($F_{Acc}^{FRET}$) and the other due to direct excitation of the acceptor ($F_{Acc}^{Direct}$):

$$F_{Acc}=F_{Acc}^{FRET}+F_{Acc}^{Direct}.$$ (1)

The control protein that contains TnC-Acceptor only (TV-Link) allows estimation of $F_{Acc}^{Direct}$ at each λ_ex_ when normalized for fluorescence intensity obtained by recording peak acceptor emission due to maximal, direct excitation of the acceptor (λ_ex_ = 515nm for mVenus or cpVenus, and λ_ex_ = 506nm for mNeonGreen) for each condition tested. To obtain $F_{Acc}^{FRET}$, according to Eq 1, $F_{Acc}^{Direct}$ was subtracted from *F*_*Acc*_. After all corrections were applied, a fluorescence ratio (F_R_) was calculated for each λ_ex_:

$$F_{R}=\frac{F_{Acc}^{FRET}}{F_{Don}}.$$ (2)

F_R_ is an indicator of FRET, because when FRET is enhanced, $F_{Acc}^{FRET}$ increases while F_Don_ decreases. This was repeated for each spectrum in each condition (Fig 2B).

### Analytical Ultracentrifugation (AUC)

AUC was used to investigate conformational changes associated with divalent cation binding to CTV-TnC WT and EF-hand mutants. Protein of interest was split into 3 samples and each was dialyzed against a different buffer: Buffer I (50mM MOPS pH = 7.0, 0.1M KCl, 3mM CaCl_2_)_,_ Buffer II (50mM MOPS pH = 7.0, 0.1M KCl, 3mM EGTA, 3mM MgCl_2_), or Buffer III (50mM MOPS pH = 7.0, 0.1M KCl, 3mM EDTA). Protein was diluted in the appropriate buffer to a final concentration of 0.3–0.5mg/ml. For sedimentation velocity measurements, 420μl of sample and 440μl of water (reference) were added to an Eppon-2 Channel cell (Beckman Coulter), and spun at 42,500 rpm at 5°C in an AN-60 Ti rotor, Beckman Xl-1 centrifuge (Beckman Coulter Inc., Fullerton, CA, USA). AUC data were analyzed using UltraScan III analysis software (UltraScan Project, UTHSCSA, Department of Biochemistry, The University of Texas Health Science Center, San Antonio, TX). Data were first analyzed with 2 Dimensional Spectrum Analysis (2DSA) with simultaneous time invariant noise subtraction according to the method of Schuck and Demeler [46]. After noise subtraction, the data were examined for heterogeneity with the enhanced Van Holde-Weischet analysis [47]. Results of these analyses are reported as the sedimentation coefficient (*s*) versus the relative frequency. Each peak is fit using nonlinear least square regression to a 3 parameter Gaussian distribution:

$$f={A_{0}}^{\left(-0.5{\left(\frac{x-s}{\delta }\right)}^{2}\right)}$$ (3)

where *s* is the mean of the Gaussian distribution, δ is the standard deviation for *s*, and A_0_ is the amplitude of the peak value. We report *s* and δ obtained from this regression. In addition, we also report the frictional coefficient *f/f*_*0*_ obtained from 2DSA. *f/f*_*0*_ is a shape indicator that varies between 1 and 4, with 1 corresponding to a perfect sphere and 4 to an elongated molecule (e.g., DNA).

### Skinned Fiber Force Measurements

Permeabilized preparations from porcine ventricular muscle were obtained essentially as described [48,49]. The fiber diameter varied between 100–300μm. Measurements were performed in buffers containing: 20mM MOPS, 7mM EGTA, 20mM creatine phosphate, 15 units/ml of creatine phosphokinase, ionic strength fixed at 150mM adjusted with KProp, 2.5mM MgATP^2-^, 1mM free Mg^2+^, 10^−8^–10^-4^M free Ca^2+^, pH 7.0; composition was determined using a custom algorithm [50]. Maximum active force and passive force were measured at pCa4 and pCa8, respectively [50]. After CDTA extraction of endogenous TnC, residual Ca^2+^-activated force was measured and values < ~20% of the pre-extraction force were considered satisfactory. TnC-extracted fiber bundles were then reconstituted with either WT-TnC or CTV-TnC at 50μM in relaxing solution (20mM MOPS, 91.9mM KProp, 7mM EGTA, 10^-8^M free Ca^2+^, 1mM free Mg^2+^, 2.5mM MgATP^2-^, pH = 7.0). Following reconstitution, passive force was measured at pCa8, and this was subtracted from all subsequent measurements to obtain Ca^2+^-activated force. To calculate the percent of force recovery after reconstitution, the maximum Ca^2+^-activated force at pCa4 was normalized against P_0_ (initial force before extraction). Force recovery after reconstitution >60% relative to pre-extraction force was considered satisfactory.

To measure Ca^2+^ dependence of isometric force generation, active force at each Ca^2+^ concentration was normalized against the force of the same fiber bundle at saturating Ca^2+^ (pCa4) after reconstitution. Normalized (%) steady state force-pCa data were fit using nonlinear least squares regression (SigmaPlot version 11.0, Systat Software, San Jose, CA) to the Hill equation:

$$F=\frac{100}{\left(1+10^{\left(nH)\times (pCa-pCa50\right)}\right)}$$ (4)

In Eq 4, *n*_*H*_ refers to the Hill coefficient which is an indicator of cooperativity, and *pCa*_*50*_ refers to the pCa at which 50% of maximum Ca^2+^-activated force is obtained. 6 fiber bundles (N = 6) were used for each of the proteins tested (reconstitution with WT-TnC or CTV-TnC). Student’s *t*-test (SigmaPlot version 11.0) was used to test the significance of force recovery and Ca^2+^ sensitivity. P <0.05 was considered statistically significant. For all data from skinned fiber experiments, the error bars shown represent mean + SE.

### Full Range Ca^2+^ and Mg^2+^ Titrations

Full range titrations of Ca^2+^ and Mg^2+^ were performed for CTV-TnC to measure the affinities of divalent cation binding, for comparison with previously published values for native cardiac TnC. CTV-TnC was diluted into buffer containing: 120mM MOPS, 90mM KCl, 2mM EGTA, 5mM NTA (NitriloTriAcetic Acid) pH = 7.0. Experiments were conducted at T = 22°C. To titrate Ca^2+^ in the presence of Mg^2+^, we added ~4.4mM [Mg]_total_ to achieve 2mM [Mg^2+^] free. Small aliquots of Ca or Mg were added stepwise during these titrations, and free Ca^2+^ and Mg^2+^ concentrations were calculated as described above for fiber solutions [50]. Protein concentrations ranged from 0.02μM for reconstituted Tn complex containing CTV-TnC, to 0.5μM for CTV-TnC. Fluorescence scans were obtained using a JASCO fluorometer (FP 8300, JASCO Analytical Instruments, Easton, MD, USA) over the range of 445nm to 610nm, as for end-point titrations, therefore capturing both donor and acceptor contributions to the overall emission spectrum. PMT excitation voltage was 200-400V, depending on protein concentration.

### Analysis of Fluorescence Spectra for Full Range Titrations

Peak fluorescence values for donor *F*_*Don*_ and acceptor *F*_*Acc*_ were recorded at 475nm and 515nm, respectively. $F_{Acc}^{FRET}$ was obtained as described above, and F_R_ (Eq 2) was calculated for each [Ca^2+^] or [Mg^2+^]. Four replicate measurements were made (N = 4). F_R_ data (non-normalized) were fit using a double Hill equation when Ca^2+^ was titrated in the presence or absence of Mg^2+^ to simultaneously evaluate both high and low affinity EF hands:

$$f=Fmin+(\frac{Fmax1}{1+(10^{n1(pCa-pCa501)}}+\frac{Fmax2}{1+(10^{n2(pCa-pCa502)}}).$$ (5)

When Mg^2+^ was titrated, F_R_ data (non-normalized) were fit to a single Hill equation:

$$f=Fmin+(\frac{Fmax\;1}{1+(10^{\left(n(pMg-pMg50)\right)}}).$$ (6)

The binding affinities were estimated by using:

$$Ka=\frac{1}{10^{-pM50}}$$ (7)

where M refers to Ca^2+^ or Mg^2+^.

## Results

### End-point Titrations of FRET Constructs

**Fig 4:**
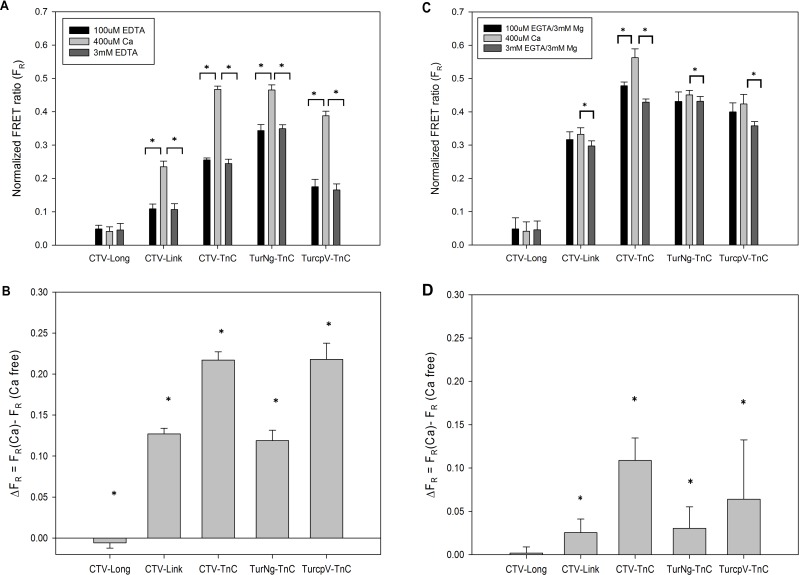
End point Ca^2+^-titrations comparing all FRET constructs in the absence or presence of 3mM Mg^2+^. In (A) the initial condition (100μM EDTA) represents apo-TnC, depleted of divalent cations, whereas in (C) the initial condition is TnC bound to ~2 Mg^2+^ achieved by adding 3mM Mg^2+^ to the starting buffer containing 100μM EGTA. Differences in F_R_ (ΔF_R_) between the Ca^2+^ saturated condition and the average initial and final conditions were calculated (B) from the data in (A) in the absence of Mg^2+^, or (D) from the data in (C) in the presence of 3mM Mg^2+^. Asterisks indicate (A and C) that bracketed values are significantly different (*t*-test, p<0.05), or (B and D) that the value is significantly different from 0 (*t*-test, p<0.01). Plotted values represent mean + SE (N = 3).

Measurements of F_R_ (Eq 2) from end-point titrations for all FRET constructs generated for this study (Fig 1) are summarized in Fig 4 in the absence (Fig 4A and 4B) or presence (Fig 4C and 4D) of physiological Mg^2+^. For panels (A) and (C) of Fig 4, each construct is represented at each of the 3 conditions tested: 100μM EDTA or Mg^2+^/EGTA (solid bars in panel A or C, respectively), 400μM Ca^2+^ (light grey bars), 3mM EDTA or Mg^2+^/EGTA (dark grey bars in panel A or C, respectively). The average of all F_R_ values at the different λ_ex_ for each divalent cation condition was calculated; error bars represent SE. Panels (B) and (D) of Fig 4 show ΔF_R_ = F_R_(Ca^2+^) - F_R_(Ca^2+^ free) calculated at each λ_ex_, and averaged across all λ_ex_. F_R_(Ca^2+^) was obtained at each λ_ex_ for the Ca^2+^ saturated condition (400μM Ca^2+^); F_R_(Ca^2+^ free) was calculated at each λ_ex_ as the average of the initial condition (100μM EDTA or Mg^2+^/EGTA in panel B or D, respectively) and the final condition (3mM EDTA or Mg^2+^/EGTA in panel B or D, respectively). The signal reported, indicative of FRET, is the FRET ratio (F_R_) (Eq 2). The advantage of using F_R_ as an indicator for FRET is that this parameter is not affected by variation in total fluorescence emission amplitude, e.g., as λ_ex_ varies (Fig 3B).

For CTV-Long, F_R_ in both the absence and presence of Mg^2+^ is small (Fig 4A and 4C, respectively), indicating that little or no FRET occurs. In addition, no consistent change (ΔF_R_) was observed upon Ca^2+^ addition (Fig 4B and 4D). We attribute these results to the presence of the long linkers between the fluorophores and cTnC (26 and 34 aa, Fig 1), rendering movement of the fluorophores uncorrelated with any conformational changes in cTnC that may occur upon divalent cation binding.

CTV-Link possesses intermediate length linkers of 8 aa each (Fig 1). The results of end point titrations for CTV-Link with Ca^2+^ are shown in Fig 4A, where no Mg^2+^ is present. We observe a fully reversible Ca^2+^ dependent change in F_R_ with amplitude of ~0.12 units (Fig 4B). For CTV-Link in the apo-TnC state, F_R_ is ~0.1 units (Fig 4A). In Fig 4C, the initial (100μM EGTA/3mM Mg^2+^) and final (3mM EGTA/3mM Mg^2+^) states are Ca^2+^ free, but are expected to have Mg^2+^ bound at the C-terminus (~2Mg^2+^ state) where F_R_ ~0.3 (Fig 4C). The higher signal in the absence of Ca^2+^ (F_R_ ~0.3 in Fig 4C compared to F_R_ ~0.1 in Fig 4A) can be explained by Mg^2+^ occupancy of sites III and IV in Fig 4C which results in a global conformational change and a corresponding increase in FRET. Addition of 400μM Ca^2+^ (saturating Ca^2+^ at all TnC sites) results in a modest increase in F_R_ of amplitude 0.05, presumably indicating a slight structural change associated with Ca^2+^ binding at N-terminal site II (Fig 4C and 4D).

CTV-TnC (Fig 1) is similar to the second construct (CTV-Link) in terms of both the fluorophores and linker length (8aa), but differs by the absence of the 5aa N-terminal extension. The magnitude of the Ca^2+^ dependent response is larger than that for CTV-Link with ΔF_R_ ~0.22 (Fig 4B). The Ca^2+^ dependent FRET change is fully reversible as can be seen in (Fig 4A). In the presence of Mg^2+^, the Ca^2+^ dependent FRET signal becomes smaller, resulting in ΔF_R_ ~0.05 unit (Fig 3C and 3D) compared to the absence of Mg^2+^. The initial and final states (no Ca^2+^ in the presence of Mg^2+^) have a higher F_R_ ~0.4 (Fig 4C) compared to the initial and final states in the absence of Mg^2+^ where F_R_ in the apo state is ~0.25 (Fig 4A). This is comparable to the results seen for CTV-Link described above, although the amplitude of the signal is larger. The Ca^2+^-dependent change in ΔF_R_ is greater when comparing the change from apo-TnC to 3Ca^2+^state of TnC (Fig 4B), versus the change from ~2Mg^2+^-TnC to 3 divalent cation state of TnC (Fig 4D). As described in the introduction, we expected that the addition of Ca^2+^ to apo-TnC would result in a significant conformational change, bringing the fluorophores in close proximity, allowing FRET to occur. When Mg^2+^ is present at the C-terminal sites, addition of Ca^2+^ that binds the N-terminus and can potentially displace the Mg^2+^ at the C-terminus results in a smaller overall conformational change. This observation is also true for construct CTV-Link, which suggests that a major conformational change occurs when divalent cations bind at the C-terminal sites.

The last two constructs, TurNg-TnC and TurcpV-TnC, have the smallest linkers (2 amino acids) between the fluorophores and cTnC (Fig 1). TurNg-TnC and TurcpV-TnC exhibit reversible Ca^2+^ dependent signals of amplitude ~0.12 and ~0.21, respectively, comparing the apo-TnC state with the 3Ca^2+^ state (Fig 3B). Both constructs show a very small Ca^2+^ dependent signal ΔF_R_ (<0.06) in the presence of Mg^2+^ (Fig 3D). This reinforces the conclusion that F_R_ signal upon divalent cation binding to the two C-terminal sites is larger than that of Ca^2+^ binding to the single N-terminal site, possibly due to larger conformational changes when the C-terminus is occupied by divalent cations.

In summary, all constructs except CTV-Long show a divalent cation-dependent FRET change. Among those that show changes in the end point titrations, CTV-Link has the smallest ΔF_R_ and F_R_ in all conditions tested. CTV-TnC and TurcpV-TnC have the largest ΔF_R_ in the absence of Mg^2+^ (Fig 4B), indicating that these constructs have the greatest FRET dependent Ca^2+^ response when comparing the apo-state to the 3Ca^2+^ state of the proteins. However, CTV-TnC shows a larger response to Ca^2+^ for end point titrations in the presence of Mg^2+^ when compared to TurNg-TnC (Fig 4D). Therefore, CTV-TnC was chosen for more detailed characterization.

### Conformational Changes Detected by AUC

**Fig 5:**
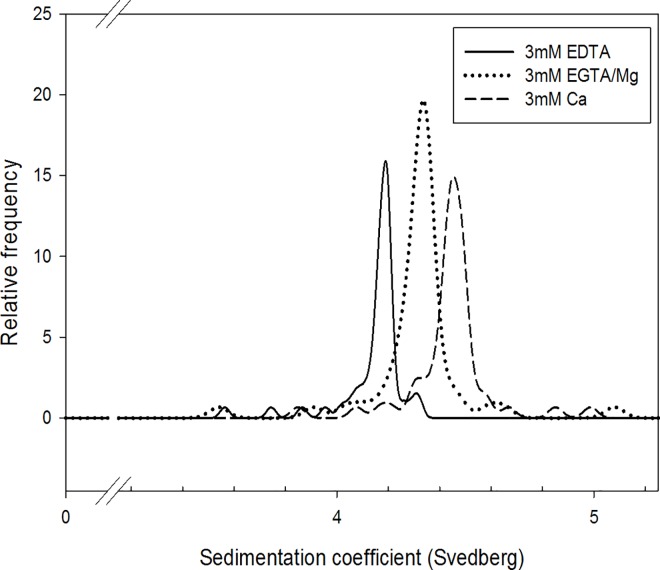
Analytical ultracentrifugation (AUC) indicates global conformational changes of CTV-TnC upon divalent cation binding. The least compact structure is apo CTV-TnC (solid line). ~2Mg^2+^-CTV-TnC (dotted line) shows an intermediate conformation, whereas Ca^2+^ saturated CTV-TnC (dashed line) adopts the most compact conformation. Sedimentation coefficients are given in the text.

We used analytical ultracentrifugation as a complementary technique to study global conformation of CTV-TnC associated with the same divalent cation states used in the end point titrations. We prepared three samples of CTV-TnC in different buffers, varying the divalent cation concentrations: 3mM Ca^2+^ (3Ca^2+^-TnC), 3mM EGTA/3mM Mg^2+^ (~2Mg^2+^-TnC) and 3mM EDTA (apo-TnC). The sedimentation coefficient (*s*) measured for CTV-TnC varies with condition (Fig 5), with the smallest *s* in the apo-state, and the largest *s* when saturated with Ca^2+^. This can be translated into global conformation. In the absence of divalent cations, CTV-TnC sediments with *s* = 4.18 + 0.03 S, and a frictional ratio *f/f*_*0*_ ~2. Upon Mg^2+^ binding at the C-terminus (~2Mg^2+^-TnC), *s* increases to 4.33 + 0.05 S, and *f/f*_*0*_ is smaller (~1.9) indicating some compaction of the protein. When it is fully saturated with Ca^2+^ (3Ca^2+^-TnC), *s* is the largest with *s* = 4.45 + 0.05 S, and *f/f*_*0*_ is the smallest (~1.7) indicating the molecule adopts the most compact structure of these three states. AUC (Fig 5) demonstrates that CTV-TnC becomes progressively more compact as the number of divalent cations bound increases from ~2Mg^2+^ at the C-terminus, to saturation with 3Ca^2+^.

### Skinned fiber mechanics

**Fig 6:**
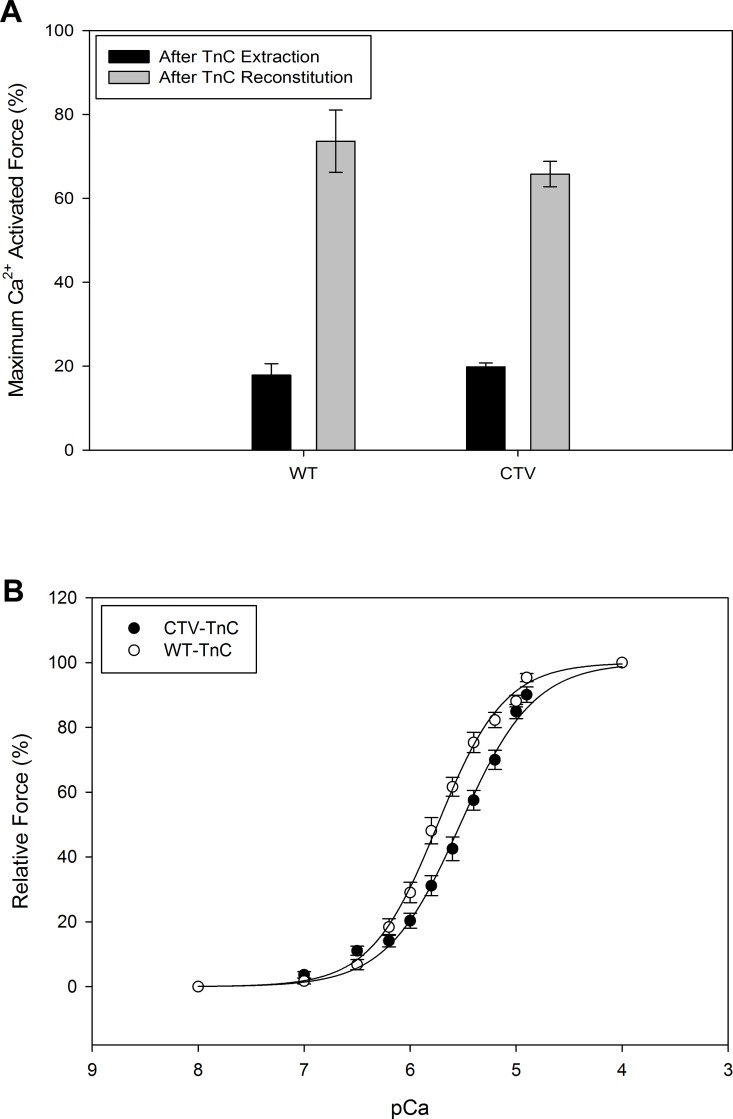
Ca^2+^-activated isometric force of skinned porcine cardiac muscle preparations reconstituted with WT-TnC or CTV-TnC. (A) Endogenous TnC was extracted (solid bars) and fibers were reconstituted with either WT-TnC or CTV-TnC (gray bars). The extent of extraction and reconstitution was defined by the percent of Ca^2+^-activated, steady state isometric force (pCa4) normalized to that measured prior to extraction of endogenous TnC. (B) Ca^2+^-sensitivity of steady state isometric force development of the two sets of reconstituted fibers was measured by incubation with different pCa solutions. Force was normalized to the maximum (pCa4) measured in the same preparation after reconstitution. Data were fit to the Hill relation (Eq 4). Plotted values represent mean + SE (N = 6).

Skinned fiber force measurements were used to test the functionality of cardiac TnC as part of the CTV-TnC protein modified by addition of two fluorophores. We compared the Ca^2+^ activated isometric force development in skinned preparations from porcine ventricular muscle from which endogenous TnC had been extracted, followed by reconstitution with WT-cTnC or CTV-TnC. Fig 6A shows the averaged (N = 6) maximum Ca^2+^ activated force (pCa4) in skinned fiber preparations after endogenous TnC extraction and following reconstitution with either WT-cTnC or CTV-TnC, normalized (%) to the maximum Ca^2+^ activated isometric force prior to extraction of endogenous TnC. The measurements for each TnC in Fig 6A define the extent of TnC extraction and reconstitution. Following extraction of ~80% of endogenous TnC (solid bars), fibers reconstituted with WT-TnC or CTV-TnC exhibited no statistically significant difference in maximum Ca^2+^-activated force (~70%; Fig 6A). Fig 6B shows the relative force (% of maximum Ca^2+^ activated force for each TnC) measured during steady-state activation over the range of pCa values which yielded a full Ca^2+^ titration. The average pCa_50_ for fibers reconstituted with WT-cTnC was 5.74 + 0.01 and 5.53 + 0.02 for CTV-TnC reconstituted fibers, resulting in a rightward shift of 0.21 pCa units with the FRET construct. The Hill coefficient *n* was 1.37 + 0.05 for fibers reconstituted with WT-cTnC and 1.25 + 0.06 for fibers reconstituted with CTV-TnC. We conclude that the FRET construct CTV-TnC can functionally reconstitute into troponin complex in thin filaments within the sarcomere lattice of fibers, and can regulate activation of cardiac muscle in a Ca^2+^ dependent manner with a slight de-sensitization to Ca^2+^.

### EF-hand mutants of CTV-TnC

**Fig 7:**
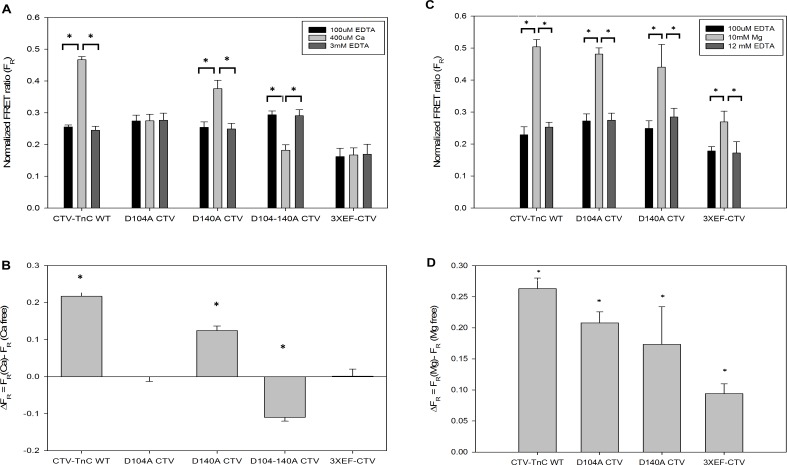
Ca^2+^ and Mg^2+^ end point titrations of EF-hand-inactivation mutants of CTV-TnC. Mutants of CTV-TnC were generated by inactivating one or more EF hands (see Materials and Methods) to separate the contribution of the different EF hands to the total FRET signal. End point titrations with Ca^2+^ in the absence of Mg^2+^ (A, B) were performed with mutants as described for CTV-TnC WT in (Fig 4A and 4B). For comparison, F_R_ and ΔF_R_ for CTV-TnC WT were replotted (in A and B, respectively) from (Fig 4A and 4B). Panel (C) shows the effects of Mg^2+^ on F_R_. (D) shows ΔF_R_ for data in C due to Mg^2+^ binding. Asterisks indicate (A and C) that bracketed values are significantly different (*t*-test, p<0.05), or (B and D) that the value is significantly different from 0 (t-test, p<0.01). Plotted values represent mean + SE (N = 3).

To separate the contribution of each cation binding site of CTV-TnC to F_R_, we evaluated a series of mutants where one or more Ca^2+^ binding site on TnC was abolished (Materials and Methods). We performed two types of end point titration: one was performed with Ca^2+^ (Fig 7A and 7B), while the second was performed with Mg^2+^ (Fig 7C and 7D). Fig 7A and 7C show F_R_ for each condition tested, whereas B and D represent ΔF_R_. For comparison, CTV-TnC WT was also included, and is the same as CTV-TnC used in experiments above.

Mutation of site III (D104A) abolished the Ca^2+^ dependent FRET signal completely which could occur either because all of the signal comes from Ca^2+^ binding at site III or else sites II and IV have equal but opposite contributions. The latter appears more likely because mutation of site IV (D140A) decreased the signal almost by half (Fig 7B). The double mutant (D104-140A) yielded a decrease, as indicated by a negative ΔF_R_ signal of -0.12. The triple mutant 3XEF-CTV does not show a Ca^2+^ dependent change; this is consistent with the expectation that when all three sites were inactivated, Ca^2+^ no longer binds to TnC and no FRET change was expected. The contribution of each site was estimated from the data in Fig 7B using a linear matrix of equations and assuming independence. The results in Table 1 (first column under F_R_), after correction with the triple mutant F_R_ signal, show that all three sites contribute to the signal, with the largest from site III and nearly equal but opposite in amplitude contributions from sites II and IV. The lower portion of the table shows the sum of all three EF hand signals after correction (F_R_ = 0.204) is similar to the signal resulting from the CTV-TnC (F_R_ = 0.217).

**Table 1:** Calculated contributions of each divalent cation binding site of CTV-TnC to F_R_, determined using EF hand mutants of CTV-TnC.

	FRET ratio (F_R_)	
TnC EF hand	Ca^2+^	Mg^2+^
II	-0.120	—
III	0.224	0.079
IV	0.100	0.114
None	0.010	0.094
Sum of 3 EF hand	0.204	0.193
CTV-TnC WT	0.217	0.169

Contributions of each site were estimated from the data in Fig 7 using a linear matrix of equations. ‘None’ refers to mutant 3XEF-CTV; this mutant provides a baseline that was subtracted from all ΔF_R_ values. For validation of our model, the predicted sum of all 3 sites in the case of Ca^2+^ (binding to all 3 EF hands), or the sum of 2 sites in the case of Mg^2+^ (binding to sites III and IV) is given in the second to last row. This prediction can be compared to the F_R_ signal obtained directly from CTV-TnC WT in the end point titration with Ca^2+^ or Mg^2+^ (last row).

The same approach was used to characterize Mg^2+^ binding by each construct and its effect on the FRET signal (Fig 7C and 7D). The double mutant D104-140A CTV was not included here because Mg^2+^ is expected to bind, at most, very weakly to the N-terminus. Mutation of site III decreases the signal by ~0.06 units compared to CTV-TnC WT, whereas site IV mutation decreases the signal by ~0.1 unit. The contribution of each site was estimated from the data in Fig 7D using a linear matrix of equations and assuming independence. Results are reported in Table 1 (second column under F_R_) after subtraction of the triple mutant F_R_ signal. The sum of the contributions of sites III and IV to the overall signal was calculated (0.193) for comparison with the signal from CTV-TnC WT (0.169) (lower part of Table 1).

Surprisingly, there was a small but statistically significant increase in F_R_ for the triple mutant at 10mM Mg^2+^, whereas saturating Ca^2+^ had a negligible effect as expected (Table 1). This result supports the possibility that very low affinity, non-EF-hand sites for Mg^2+^ binding are present as reported by others [2,51]. Potter and Gergely [52] have suggested that sTnC has two sites that bind Mg^2+^ nonspecifically, in addition to the known four EF hands. From the data in Table 1, it is clear that site III contribution to the FRET signal is different in the presence of Ca^2+^ (0.224) vs Mg^2+^ (0.079). Site IV differs only slightly with Ca^2+^ (0.100) vs Mg^2+^ (0.114). The analysis in Table 1 suggests that site III is the dominant component of the Ca^2+^-dependent FRET signal in our study. Finally, Ca^2+^ binding at site II results in a negative contribution (-0.120), reducing the overall signal. In terms of conformational changes, a positive signal could be interpreted as a compaction of TnC bringing the ends and the fluorophores closer together, whereas a negative signal could mean an opening of TnC or a rotation of the N- and C-domains relative to one another, resulting in an increase in the distance of the ends and the fluorophores; it could also mean a change in orientation of the two fluorophores relative to one another, changing their dipole coupling, rendering FRET less optimal [53].

AUC measurements were performed using mutant proteins D104A CTV, D140A CTV, and D104-140A CTV to correlate the observed changes in FRET with global conformation associated with each of the EF hands upon divalent cation binding. Similarly to CTV-TnC described above, each mutant was prepared in 3 different buffer conditions: 3mM EDTA, 3mM EGTA/3mM Mg^2+^ and 3mM Ca^2+^. Each protein’s sedimentation coefficient was compared under these 3 states, apo-state, ~2Mg^2+^ state and 3Ca^2+^ state, respectively. Table 2 summarizes the sedimentation coefficients obtained. The apo state shows very close *s* values for all mutants as can be seen in the first row of Table 2. The double mutant D104-140A CTV exhibits no significant difference in *s* between the apo-state and the 3Ca^2+^ state, indicating that the negative FRET signal induced by Ca^2+^ binding to the N-terminus alone (Fig 7, Table 1) does not appear to correlate with a conformational change in the AUC data (Table 2). The ~2Mg^2+^-state shows a moderate yet significant increase in *s* relative to the apo state for all three mutants. From data for mutant D140A CTV, site III shows the largest FRET signal in the presence of Ca^2+^ compared to Mg^2+^, paralleled by a larger sedimentation coefficient for Ca^2+^ (4.108 + 0.048) than for Mg^2+^ (4.036 + 0.080). Similarly, data for mutant D104A CTV show that site IV confers a larger FRET signal in the presence of Mg^2+^ compared to Ca^2+^, consistent with a larger sedimentation coefficient (4.107 + 0.031) and (4.070 + 0.012) respectively. These AUC results are in agreement with the end point titration experiments summarized in Table 1. Because binding of Mg^2+^ versus Ca^2+^ at sites III and IV affect FRET in a different manner, different global conformations detected by AUC are induced in cTnC. In addition, for mutant D104-140A with only site II active, Mg^2+^ induces a change in sedimentation coefficient compared to apo and Ca^2+^-bound TnC (Table 2). This was surprising because mutant D104-140A has sites III and IV inactivated. Braga et al. [54] showed that low affinity Mg^2+^ binding occurs at the C-terminus of sTnC, distinct from the two higher affinity Ca^2+^/Mg^2+^ binding sites. They also showed that increasing Mg^2+^ concentration in skinned fibers (10-44mM) led to dissociation of TnC from thin filaments, which can be explained by a conformational change of TnC. Taken together, these observations could account for the change in AUC sedimentation coefficient upon non-physiological Mg^2+^ binding even in the absence of active sites III and IV.

**Table 2:** Summary of sedimentation coefficients (*s*) for EF hand mutants of CTV-TnC: D104A CTV, D140A CTV, D104-140A CTV.

Sedimentation coefficient (*s*)	D104A CTV	D140A CTV	D104-140A CTV
Apo state	3.937 + 0.019	3.957 + 0.041	3.918 + 0.018
Mg^2+^ bound state	4.107 + 0.031*	4.036 + 0.080*	3.991 + 0.020*
Ca^2+^ bound state	4.070 + 0.012*	4.108 + 0.048*	3.930 + 0.019

* p<0.01 Mg^2+^ or Ca^2+^ bound state *vs* Apo state within the same protein.

Values represent mean + S.E.

### Full Range Titrations of CTV-TnC and CTV-Tn with Ca^2+^ and/or Mg^2+^

**Fig 8:**
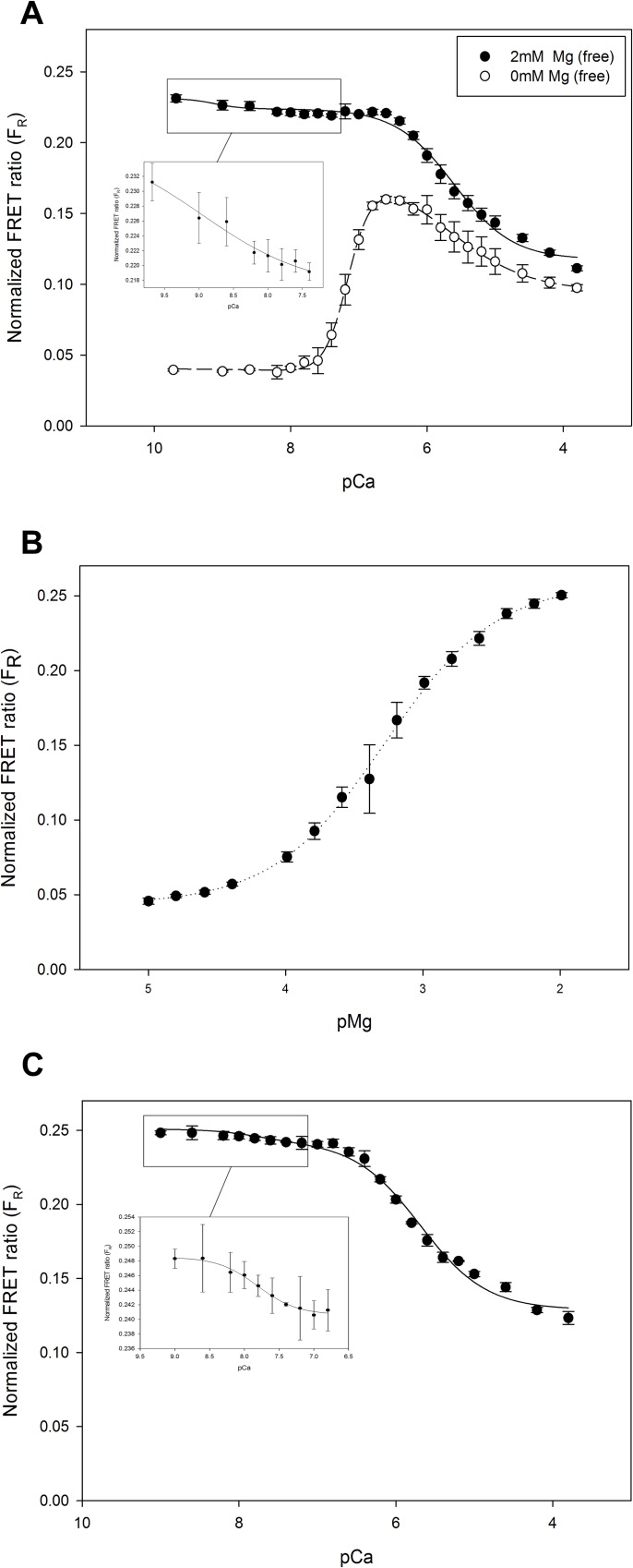
Ca^2+^ and Mg^2+^ titrations of CTV-TnC and CTV-Tn complex allow determination of EF hand affinities for divalent cations from F_R_. (A) Ca^2+^ titrations of CTV-TnC (1μM) were performed in the presence (solid symbols) or absence (open symbols) of 2mM Mg^2+^_free_ to determine the affinities of sites III and IV (high affinity), and site II (low affinity) for Ca^2+^. (B) Mg^2+^ titration of CTV-TnC (1 μM), where the binding of Mg^2+^ is shown for sites III and IV. (C) Ca^2+^ titration of CTV-TnC reconstituted in Tn complex, in the presence of 2mM Mg^2+^_free_. Insets in (A) and (C) show expansion of the initial portion of the titrations for clarity. Data were fitted using nonlinear least squares regression to either a double-Hill relation (Eq 5) in (A and C), or a single Hill relation (Eq 6) in (B). All regression parameter estimates are given in Table 3. Plotted values represent mean + SE (N = 4).

We titrated the single protein CTV-TnC with Ca^2+^ in the absence of Mg^2+^ (Fig 8A, open circles) or in the presence of ~2mM Mg^2+^ free (Fig 8A, closed circles). It is worth noting that as we titrated the high affinity sites which presumably correspond to the C-terminus of TnC (sites III and IV), F_R_ amplitude increases. On the other hand, titrating the low affinity site expected to correspond to the N-terminus of TnC (site II) results in decrease of F_R_. Based on the end-point titration data in Fig 4, it was surprising that the full titrations with Ca^2+^ revealed a biphasic response with larger overall amplitude, although the biphasic response and assignment of FRET changes to C- and N-domain sites are consistent with our analysis of the EF hand mutant data (Table 1). The data in Fig 8A were fit with a double Hill equation (Eq 5) to describe the biphasic response in a manner consistent with the known existence of two classes of divalent cation binding sites—high and low affinity sites in the C- and N-domains, respectively. Regression parameter estimates are summarized in Table 3. All parameters with subscript _1_ refer to the first component which corresponds to higher affinity binding; likewise, parameters with subscript _2_ refer to the second component which corresponds to lower affinity binding. In the absence of Mg, *pCa*_*50* 1_ is 7.17 (Fig 8A, open symbols, and Table 3), but it increases to 9.1 in the presence of 2mM Mg^2+^ (Fig 8A, closed symbols, inset, and Table 3). Our analysis assumes that the affinities of sites III and IV are indistinguishable. pCa_50 2_ was 5.47 in the absence of Mg^2+^ (Fig 8A, open symbols, and Table 3) and increases to 5.60 in the presence of Mg^2+^ (Fig 8A, closed symbols, inset, and Table 3). The Hill coefficient for the high affinity sites *n*_*H 1*_ is ~2 for the Ca^2+^ titration both in the presence and absence of 2mM free Mg^2+^, whereas *n*_*H 2*_ corresponding to the low affinity sites is ~1 in both titrations (Table 3). This is in agreement with the interpretation that high affinity binding corresponds to divalent cation binding at two sites in the C-domain, and low affinity binding corresponds to one site in the N-domain. Data also suggest that in the presence of Mg^2+^ at the C-terminal sites, addition of Ca^2+^ that binds the N-terminus and displaces the Mg^2+^ at the C-terminus (Fig 8A), which is in agreement with findings by others [18,55].

**Table 3:** Summary of regression parameter estimates from Hill equation fits of the Ca^2+^ and/or Mg^2+^ titrations of CTV-TnC and CTV-Tn complex in Fig 8.

	CTV-TnC			CTV-Tn complex
Parameters	Ca^2+^ titration 0mM Mg	Ca^2+^ titration 2mM Mg	Mg^2+^ titration	Ca^2+^ titration 2mM Mg
*F*_*min*_	0.040 ± 0.001	0.231 ± 0.002	0.043 ± 0.003	0.251 ± 0.006
*pCa*_*50 1*_/*pMg*_*50*_^#^	7.17 ± 0.06	9.12 ± 1.12 *	3.28 ± 0.10	7.81 ± 0.05 *
*n*_*H 1*_	2.70 ± 0.30	2.02 ± 0.96 *	1.09 ± 0.08	1.53 ± 0.28 *
*F*_*max 1*_	0.133 ±0.006	-0.008 ± 0.012 *	0.215 ± 0.006	-0.008 ± 0.002 *
*pCa*_*50 2*_	5.47 ±0.38	5.60 ± 0.12	—	5.69 ± 0.04
*n*_*H 2*_	0.94 ± 0.50	1.07 ± 0.07	—	1.04 ± 0.09
*F*_*max 2*_	-0.082 ± 0.018	-0.106 ± 0.002	—	-0.114 ± 0.004

* Indicates that the error represents a standard error associated with the fit parameters; all other errors represent +SD

^#^Indicates pMg_50_ measured upon “Mg^2+^ titration”

F_max_ is the amplitude of the maximum, divalent cation dependent change in F_R,_ *F*_*min*_ is the value of F_R_ signal at low concentrations of the titrant divalent cation, *n*_*H*_ is the Hill coefficient, *pCa*_*50*_ is the pCa value at which 50% of the signal change is achieved, *pMg*_*50*_ is the pMg value at which 50% of the signal change is achieved.

Similarly, a full range Mg^2+^ titration of CTV-TnC was performed (Fig 8B). Data were fit to a single Hill equation (Eq 6), yielding *pMg*_*50*_ of 3.28, which we expect to correspond to the Mg^2+^ affinity of C-terminal sites III and IV. Regression parameter estimates are summarized in Table 3.

Finally, Ca^2+^ titration was also performed with Tn complex reconstituted with CTV-TnC (Fig 8C). These experiments require the presence of Mg^2+^ to maintain proper complex assembly, hence titrations of Mg^2+^ or titrations of Ca^2+^ in the absence of Mg^2+^ were not attempted. In an independent experiment to further confirm that CTV-TnC can reconstitute into isolated Tn complex (with TnI and TnT), we assayed the assembled complex by non-denaturing gel electrophoresis and size exclusion chromatography (data not shown). Data in Fig 8C were fit using the double Hill equation (Eq 5) resulting in *pCa*_*50* 1_ of 7.81 and *pCa*_*50* 2_ of 5.69 (Table 3). Regression parameter estimates are given in Table 3.

## Discussion and Conclusion

We designed five potential Ca^2+^dependent FRET indicators using the FRET pair of fluorescent proteins CFP and YFP attached at either end of human cardiac TnC to form fusion proteins (Fig 1). Our purposes include generation of genetically encoded Ca^2+^ indicators, and also investigation of structural changes in TnC upon divalent cation binding, particularly the global movement of the N-terminus relative to the C-terminus that determines compactness of the molecule. The construct CTV-TnC was selected for detailed analysis. Affinities of TnC for Ca^2+^ and Mg^2+^, derived from FRET signals of the novel construct, are in reasonable agreement with published values for TnC alone [2,56]. Our results are consistent with a global compaction of cTnC as it binds divalent cations in solution, with similar changes occurring within the Tn complex.

### Fluorescent protein constructs of cTnC report on global cTnC structure

Four FRET constructs exhibited an increase in F_R_ upon Ca^2+^ binding which was fully reversed upon Ca^2+^ removal (Fig 4). Higher values of F_R_ were also associated with Mg^2+^ binding (Fig 4). The divalent cation dependent FRET changes were associated with changes in conformation as demonstrated by AUC for CTV-TnC (Fig 5). CTV-TnC was able to reconstitute into Tn complex in solution and in skinned fibers (as assessed by non-denaturing gel electrophoresis, size exclusion chromatography (SEC) and maximal force recovery). pCa_50_ for fibers reconstituted with CTV-TnC was 5.53 + 0.02 (K_a_ = 3.4 x 10^5^ M^-1^) whereas pCa_50_ for fibers reconstituted with WT-cTnC was 5.74 + 0.01 (K_a_ = 5.5 x 10^5^ M^-1^). The rightward shift in Ca^2+^ responsiveness, although non-negligible in physiological terms, is similar to that observed with a number of single amino acid changes in troponin or tropomyosin, and is smaller than the most extreme shifts in pCa_50_ associated with single residue mutants [43,48,57,58] or short truncations of the N-terminus [59]. We also performed independent titrations in solution with CTV-TnC alone (Fig 8A and 8B) or as part of Tn complex (Fig 8C). Affinities for Ca^2+^ and Mg^2+^ (Table 3) are in close agreement with published values for sites III/IV and site II respectively [2,56]. Taken together, these results indicate that the fluorescent proteins have a remarkably small impact on TnC function—taking into account the large modifications to cTnC by adding two fluorescent fusion proteins—and thus report on structural changes upon metal ion binding.

### Structural changes in cTnC upon divalent cation binding

End point titrations of Ca^2+^ in the absence or presence of Mg^2+^ for the four constructs that responded to divalent cation binding indicate that Ca^2+^ dependent ΔF_R_ is larger in the absence of Mg^2+^ (Fig 4).

CTV-TnC shows compaction as more divalent cations bind progressively to cTnC, as demonstrated by FRET increase as Ca^2+^ binds (Fig 4), corresponding to more compact conformations upon divalent cation binding demonstrated by AUC (Fig 5). This compaction can be compared with existing, high resolution structures of TnC. The N- and C-domains of sTnC consist of two globular domains separated by a long, well defined, nine-turn α-helical central DE-helix in crystal structures of avian sTnC at low pH, where ions occupy only the C-domain and the N-domain is unoccupied [12,13]. In addition, the N- and C-domains of sTnC are closer, with a collapsed central DE-helix in the crystal structure of Mg^2+^-saturated sTn core domain [6]. Thus the divalent cation-dependent compaction that we observe in solution appears to contrast with the elongation of sTnC in sTn’s core domain [6], although sTnC can retain an elongated structure when the N-domain is unoccupied [12,13]. Even though our approaches do not directly report on the structure of the central DE-helix, the flexibility of the central linker is inferred from FRET data. Our observations on cTnC in solution are more compatible with the collapsed central DE-helix in the crystal structure of Ca^2+^-saturated cTn core domain [7]. There is not yet a corresponding structure for comparison with only the C-domain occupied. NMR solution structures of Ca^2+^-saturated sTnC and cTnC indicate that the central DE-helix of both molecules is highly flexible [15,60,61]. Our results indicate that, even with a highly flexible linker, the relationship between the N- and C-domains is determined by divalent cation occupancy.

Structure of the TnC linker may be different between: i) crystal and solution structures for TnC; ii) cardiac and skeletal TnC both for TnC; iii) in the Tn core domain crystal structures. Structural studies show that TnC structure appears to be dependent not only on Ca^2+^ presence and isoforms, but also the presence of other Tn subunits [18,19,62]. In the present study, we show from titration studies (Fig 8A and 8B) that CTV-TnC alone and as part of the Tn complex behaves similarly as Ca^2+^ is added in the presence of physiological Mg^2+^. Our results suggest that the global structure of cTnC undergoes similar changes upon divalent cation binding in the absence or presence of other Tn subunits, but this observation need to be further investigated to be confirmed. Interestingly, Sevrieva et al. [35] show that cTnC in the cardiac sarcomere undergoes a compaction and rotation when Ca^2+^ binds to site II. Comparable structural changes were reported in molecular dynamics simulations [63] that suggest the main structural changes are driven by ordering of the ends of cTnC’s central helix in response to divalent cation binding. This mechanism not only implies that cTnC is central to the divalent cation dependent structural changes in cTn, but also provide a mechanism by which the ends of the central linker contribute to how TnC distinguishes between monodentate coordination of Mg^2+^ and bidentate coordination of Ca^2+^ [4,61].

### Divalent cation affinities of EF hands in CTV-TnC

To determine the affinities of CTV-TnC to divalent cations we titrated CTV-TnC first in the absence of Mg^2+^ (Fig 8A) which revealed both high Ca^2+^ affinity binding of K_a_ = 1.5 x 10^7^ M^-1^ (pCa_50_ = 7.17), and lower Ca^2+^ affinity binding site of K_a_ = 3 x 10^5^ M^-1^ (pCa_50_ = 5.47) (Table 3). Even though initial F_R_ (at low Ca^2+^) was elevated in the presence of 2mM free Mg^2+^, as expected (Fig 4), we could still detect changes in F_R_ as Ca^2+^ replaced Mg^2+^ at the higher affinity C-terminal sites (Fig 8A inset); one possible explanation is that the global structure of TnC, and thus F_R_, is slightly different with Ca^2+^ versus Mg^2+^ bound at sites III and IV, in agreement with antibody-detected changes in TnC structure Jin et al. [11], and as could be expected from the monodentate coordination of Mg^2+^ compared with the bidentate coordination of Ca^2+^ [4,61,64] that could alter the structure of not just the local domain structure but also the structure of the central helix [63]. The apparent affinity constant for the high affinity sites becomes close to 1.3 x 10^9^ M^-1^ (pCa_50_ = 9.1 + 1.1); the relatively large error in this regression parameter estimate can be attributed to the small amplitude of change in F_R_ (F_max1_ in Table 3). As part of the Tn complex, CTV-TnC affinities for Ca^2+^ in the presence of Mg^2+^ are also in reasonable agreement with published values for Tn complex in solution. We estimated K_a_ = 6.5 x 10^7^ M^-1^ (pCa_50_ = 7.81) for the high affinity sites, and K_a_ = 4.9 x 10^5^ M^-1^ (pCa_50_ = 5.69) for the low affinity site (Fig 8C, and Table 3). The solution data are also in agreement with our affinity estimates for Ca^2+^ binding at site II from the fiber studies (Fig 6), although it is worth noting that force provides only an indirect measure of Ca^2+^ binding. Modest differences in affinity are expected for TnC in increasingly complex systems such as Tn complex and thin filaments, due to changes in kinetics of Ca^2+^ binding and dissociation [65]. Finally, Mg^2+^ titration yielded Mg^2+^ affinity of K_a_ = 1.9 x 10^3^ M^-1^ (pMg_50_ = 3.28) (Fig 8B, and Table 3) consistent with the K_a_ reported by Johnson et al. [2] of 2 x 10^3^ M^-1^ for C-terminal sites III/IV, which are the only sites to bind Mg^2+^ with such an affinity. Although C-terminal sites III and IV are commonly considered to be predominantly occupied by Mg^2+^, the implication of our measurements (Fig 8 and Table 3) for cardiomyocyte physiology is that a substantial fraction of the C-terminal sites are occupied by Ca^2+^ at all times, even in diastole. If this is in fact the case, it may have significant implications for cardiomyocyte function [55].

### Contribution of high affinity Ca^2+^ or Mg^2+^ binding to global structural changes in TnC

C-terminal sites III and IV are generally treated as indistinguishable both in terms of affinities to divalent cations (as was the case for our analysis of CTV-TnC WT), and in terms of structural changes induced upon binding divalent cations. Sites III and IV are considered as structural sites because of their fundamental role in anchoring TnC to the thin filament, while the regulation of muscle function occurs at site II, which has been the primary target of most studies. A key, novel finding in our study is that each of these sites makes an essentially independent contribution to the global conformation to TnC upon binding divalent cations (Table 1). We mutated sites III and IV, one at a time, and both together, and studied the mutants by end point titrations and AUC. Site III shows a higher FRET signal when bound to Ca^2+^ compared to Mg^2+^ (ΔF_R_ 0.224 versus 0.079, respectively), derived from experiments with mutant D140A CTV (site IV inactivated). In addition, AUC data for the same mutant show a larger *s* in the presence of Ca^2+^ compared to Mg^2+^ (Table 2). We conclude that Ca^2+^ binding to site III induces a larger global conformational change in cTnC, making the molecule more compact when compared to Mg^2+^ binding to this site. Conversely, as demonstrated by mutant D104A CTV where site III is inactivated, Mg^2+^ binding to IV induces a larger FRET signal then when bound to Ca^2+^ (ΔF_R_ 0.114 versus 0.100, respectively) (Table 1). Concurrently, AUC data shows that sedimentation coefficient for this mutant is larger bound to Mg^2+^ in comparison to Ca^2+^. Our FRET and AUC analyses of the CTV-TnC mutants show that sites III and IV are distinct because they induce different conformational changes in TnC when bound to Ca^2+^ versus Mg^2+^, and thus provide an explanation for the changes in F_R_ observed in WT when Ca^2+^ displaces Mg^2+^ at sites III and IV (Fig 8A and 8C).

### Contribution of low affinity Ca^2+^ binding to structural changes in TnC

For all conditions examined, the lower affinity (*pCa*_*50 2*_) and *n*_*H 2*_ ~1 are consistent with Ca^2+^ binding at site II of cTnC, and were associated with a decrease in F_R_ (negative ΔF_R_ or F_max2_) (Figs 7 and 8, Tables 1 and 3). While this could be due to an increased distance between the donor and acceptor, AUC measurements indicated otherwise. CTV-TnC WT becomes more compact when Ca^2+^ displaces Mg^2+^ at sites III and IV, and binds all three EF-hands (Fig 5). In contrast, there was no change in *s* for D104-140A between the apo and Ca^2+^-bound states, and therefore no significant structural changes when Ca^2+^ binds at site II alone (Table 2). Decrease in F_R_ could alternatively be related to a change in fluorescence lifetime of one or both of the fluorophores upon Ca^2+^ binding to site II. Laine et al. [53] showed that two constructs, TN-L15 and mTFP-TnC-Cit similar in design to our constructs, exhibited a decrease in fluorescence lifetime of the donor upon Ca^2+^ binding. Another possibility is that Ca^2+^ binding to site II, when sites III and IV are occupied, results in a change in orientation of the probes and dipole coupling, resulting in a decreased FRET signal. This would be consistent with the rotation of cTnC’s N-domain predicted by molecular dynamics [63] and observed in the intact sarcomere [35], suggesting that the changes observed in more complex systems could be driven by the divalent cation-dependent, global structural changes in cTnC (Fig 7).

### Implications for muscle biochemistry and biophysics

We have demonstrated that CTV-TnC responds to divalent cation binding through a change in global conformation, allowing energy transfer. Ca^2+^ sensors (small molecule and engineered proteins) have been used to follow cellular Ca^2+^ dynamics in live cells. CTV-TnC presents great potential to be used as a Ca^2+^ sensor, as part of the family of GECI’s. The advantage of GECIs includes their ability to be targeted to specific organelles in a cell, and could also be potentially delivered by injection of the protein directly into cells/tissues. Additionally, TnC is only expressed in muscle tissues and therefore is less likely to interfere with the function of most cells, as opposed to calmodulin (used in some GECI’s) which may deregulate signaling [66]. We acknowledge that the use of our sensor in non-muscle systems will require further investigation and characterization.

Furthermore, our Ca^2+^ sensor is of particular interest to muscle research. To our knowledge, this construct is the first GECI FRET reporter of divalent cation binding to full length cardiac TnC while retaining its function to bind and activate the myofilament, albeit with a relatively small, non-negligible Ca^2+^ desensitization. This allows our sensor to have numerous potential applications such as live myocyte imaging, and could also be used to study thin filament dynamics since it was designed to be a single molecule reporter [67]. While a considerable amount of information is available about TnC structure and function, most data are derived from ensemble measurements. Therefore, single molecule studies would greatly enhance our understanding of relationship between the structural dynamics of TnC and thin filament function [68], and onset of diseases involving thin filament protein mutations.
